# Encapsulating Azolates Within Cationic Metal–Organic Frameworks for High‐Energy‐Density Materials

**DOI:** 10.1002/advs.202409093

**Published:** 2024-09-27

**Authors:** Ning Ding, Chaofeng Zhao, Jichuan Zhang, Yao Du, Qi Sun, Shenghua Li, Siping Pang

**Affiliations:** ^1^ School of Materials Science & Engineering Beijing Institute of Technology Beijing 100081 China

**Keywords:** azolate, cationic metal‐organic framework, counter anion, energetic materials, general synthesis

## Abstract

Despite the synthesis of numerous cationic metal‐organic frameworks (CMOFs), their counter anions have been primarily limited to inorganic Cl^−^, NO_3_^−^, ClO_4_^−^, BF_4_^−^, and Cr_2_O_7_^2−^, which have weak coordination abilities. In this study, a series of new CMOFs is synthesized using azolates with strong coordination abilities as counter anions, which are exclusively employed as ligands for coordinating with metals. Owing to the unique nitrogen‐rich composition of azolates, the CMOFs demonstrate significant potential as high‐energy‐density materials. Notably, CMOF(CuTNPO) has an exceptionally high heat of detonation of 7375 kJ kg^−1^, surpassing even that of the state‐of‐art CL‐20 (6536 kJ kg^−1^). To further validate the advantages of employing azolates as counter anions, analogues with azolates serving as ligands are also synthesized. The comparison study indicates that encapsulating azolates within the cationic frameworks confers both high energy and safety properties. X‐ray data and quantum calculations indicate that their enhanced performance stems from stronger H─bonds and π–π interactions. This study introduces new roles for azolates in MOFs and expands possibilities for structural diversity and potential applications of framework materials.

## Graphical abstract

In this work, the first encapsulation of multi‐nitro heterocyclic molecules is reported within a metal‐organic framework (MOF) as balanced ions. This encapsulation demonstrates enhanced energy and safety compared to analogous coordinated structures, highlighting the potential of MOFs in stabilizing complex molecular systems for various applications.

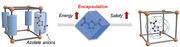

## 1 Introduction

**Figure 1:**
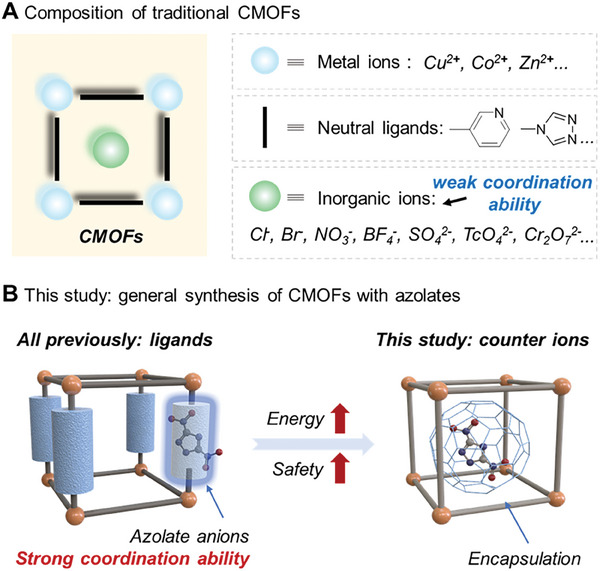
A) Composition of traditional cationic metal–organic frameworks (CMOFs); B) (left) CMOFs based on azolate anions as ligands (traditional work); (right) CMOFs based on azolate anions as charge‐balancing anions (this work).

Cationic metal‐organic frameworks (CMOFs) are an emerging class of polymeric crystalline materials. They have attracted significant attention owing to their potential applications in anion exchange and separation,^[^ ^1^ ^]^ catalysis,^[^ ^2^ ^]^ drug delivery,^[^ ^3^ ^]^ electrical conductivity,^[^ ^4^ ^]^ and energetic materials.^[^ ^5^ ^]^ Fundamentally, their structures comprise three parts: metal cations, neutral organic ligands, and uncoordinated counter anions.^[^ ^6^ ^]^ Their structures and properties can be altered by selecting certain metal ions or organic ligands and by changing the counter anions.^[^ ^7^ ^]^ Currently employed counter anions are generally limited to traditional inorganic anions (**Figure** **1**A), such as Cl^−^, Br^−^, I^−^, NO_3_^−^, ClO_4_^−^, BF_4_^−^, SO_4_^2−^, TcO_4_^2−^, SeO_4_^2−^, ReO_4_^2−^, and Cr_2_O_7_^2−^.^[^ ^8^ ^]^ This is because these anions show relatively weak coordination ability with metal nodes, which has greatly limited the further development of CMOFs.

Azoles are five‐membered, nitrogen‐containing heterocycles, such as imidazole, pyrazole, triazole, and tetrazole, whose structures contain C─H and N─H sites.^[^ ^9^ ^]^ These sites can be easily functionalized by various groups, such as ─NH_2_, ─NO_2_, ─CF_3_, ─Br, ─CH_3_, and ─NHNO_2_, rendering them excellent choices for metal–organic framework (MOF) construction.^[^ ^10^ ^]^ For example, ZIF‐8,^[^ ^11^ ^]^ which is based on imidazole, has been extensively studied for water pollution treatment, and ZnF(TZ),^[^ ^12^ ^]^ which is based on triazole, has been used for CO_2_ adsorption and separation. DBX‐1,^[^ ^13^ ^]^ which is based on tetrazole, possesses a high detonation performance and is a promising high‐energy‐density material with essential roles in solid propellants, space exploration, and defense technology. However, multiple strongly coordinating nitrogen atoms on the azole unit (e.g., two for pyrazole, three for triazole, and even four for tetrazole) result in MOFs (or even polymers) with azoles as the ligands rather than as the uncoordinated counter anions (Figure 1B).^[^ ^14^ ^]^ To the best of our knowledge, CMOFs with azolates as counter anions have not been reported. This is because the coordination interactions between metal ions, organic ligands and azolate anions are hard to precisely control and thus, encapsulating azolates within CMOFs is a significant challenge.

Herein, we present a general one‐pot synthesis of a series of CMOFs featuring six azolate anions (3,4,5‐trinitropyrazolate, 3,5‐dinitrotetrazolate, 5‐nitrotetrazolate, and their *N*‐oxides) and four metals (Fe, Co, Cu, and Zn). Their structures and potential applications as high‐energy‐density materials were investigated. To confirm the superiority of employing azolates as counter ions, an analog with azolates serving as ligands was also synthesized. The aim of this study is to enrich the structural diversity of CMOFs with unique performances and different potential applications.

## 2 Results and Discussion

### 2.1 Synthesis of the Model CMOF(CuDNT)

**Figure 2:**
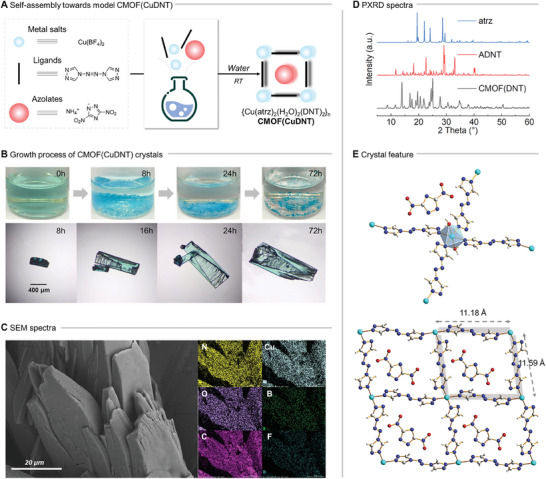
Synthesis and structural characterization of the model CMOF(CuDNT). A) Synthesis of CMOF(CuDNT); B) Growth process of CMOF(CuDNT); C) SEM images of CMOF(CuDNT); D) PXRD patterns of atrz, ADNT, and CMOF(CuDNT); E) Crystal structure and packing diagram of CMOF(CuDNT).

The synthesis of CMOFs using azolates as counter anions was investigated, and the results are shown in **Figure** **2**A. 4,4′‐Azotriazole (atrz) was used as the organic ligand because it contains eight nitrogen atoms, exhibits strong coordination with transition metals, and may inhibit the coordination of transition metals with azolate anions. For the synthesis of the model CMOF, 3,5‐dinitrotriazolate (DNT^−^) from ammonium 3,5‐dinitrotriazolate (ADNT) was selected as the typical counter anion. This is mainly because of three reasons: 1) similar to atrz ligand, DNT^−^ is also a triazole, facilitating the control of coordination ability; 2) DNT^−^ contains two strong electron‐withdrawing nitro groups, which can reduce the electron density of the N atoms on the triazole ring, and diminish the coordination ability to a certain extent; and 3) nitro groups can increase the density and oxygen balance, rendering DNT^−^ a highly energetic unit and the obtained CMOF a potential high‐energy‐density material.^[^ ^15^ ^]^ Cu(BF_4_)_2_ was selected as the typical metal source owing to the strong coordination ability of Cu^2+^ with nitrogen atoms. Moreover, BF_4_^2−^ contains neither nitrogen nor oxygen atoms, thereby contributing to the structural characterization of the target CMOF.

The optimized synthesis conditions (see supporting information) were as follows: 3 equiv. of Cu(BF_4_)_2_ was dissolved in water, which was added to a water solution containing 1 equiv. of atrz and 3 equiv. of DNT^−^. Under static ambient conditions at room temperature, dark blue plate‐like crystals of [Cu(atrz)_2_(H_2_O)_2_(DNT)_2_]_n_, CMOF(CuDNT)} were obtained after 72 h (Figure 2B). The products were highly stable in air and insoluble in water and common organic solvents. Elemental analysis, infrared (IR) spectroscopy, and mapping measurements confirmed the presence of Cu^2+^, atrz, and DNT^−^ and the absence of BF_4_^−^ (Figure 2C). Furthermore, powder X‐ray diffraction (PXRD) and scanning electron microscopy (SEM) analyses revealed that CMOF(CuDNT) had good crystallinity (Figure 2D) and that the morphology was different from those of atrz and ADNT. The gram‐scale synthesis of CMOF(CuDNT) resulted in a 1.92 g product with a high yield of 85% within 72 h (see supporting information).

To further confirm that DNT^−^ was encapsulated within the CMOF, the molecular structures of CMOF(CuDNT) were investigated using single‐crystal x‐ray diffraction. CMOF(CuDNT) crystallized in a triclinic system with space group P‐1(2). As shown in Figure 2E, each Cu^2+^ exhibited a six‐coordinate octahedral geometry with an N_4_O_2_ donor set. Four different atrz molecules were bonded to four nitrogen‐coordinating sites, while the remaining two coordinating sites were occupied by two axial water molecules. Each atrz molecule functioned as a bidentate ligand, bridging adjacent Cu^2+^ to extend the framework into a 2D layer with a repeating [Cu_4_(atrz)_4_(H_2_O)_2_] framework in the *bc* plane (Figure 2E). The [Cu_4_(atrz)_4_(H_2_O)_2_] framework featured a parallelogram window with dimensions of 11.177 × 11.588 Å^2^, within which DNT^−^ was located and uncoordinated. The shortest Cu^2+^…DNT^−^ distance was 5.26 Å (Cu…O), which was longer than the sum of the van der Waals radii (r_Cu+O_: 2.94 Å), indicating the absence of anion coordination in CMOF(CuDNT). Notably, the DNT^−^ in CMOF(CuDNT) were highly ordered within the framework structure, a feature that distinguished them from most reported CMOFs, which typically contain disordered charge‐balancing anions that are difficult to define.

### 2.2 General Synthesis and Structural Features

**Figure 3:**
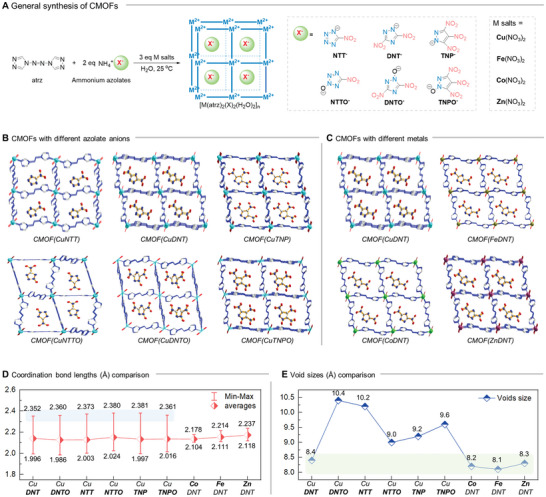
General synthesis and structural features of CMOFs. A) Synthesis of different CMOFs; B and C) Packing structures of energetic CMOFs with different azolate anions and metals; D) Comparison of coordination bond lengths for different energetic CMOFs; E) Comparison of void sizes for energetic CMOFs.

The universality of azolates and metals was then investigated. For the azolates (**Figure** **3**A,B), the successful synthesis of the model CMOF(CuDNT) showed that the nitro group facilitates the encapsulation of azole anions, based on which we extended DNT^−^ to 5‐nitrotetrazolate (NTT^−^) with one nitro group and four *N*‐coordinated sites, and 3,4,5‐trinitropyrazolate (TNP^−^) with three nitro groups and two *N*‐coordinated sites. The results indicated that these nitro‐substituted azolates were also successfully encapsulated within the cationic framework under the same synthetic conditions. In addition, the synthesis method was also effective for the *N*‐oxides of nitroazolates, including 5‐nitrotetrazolate‐2*N*‐oxide (NTTO^−^), 3,5‐dinitro‐1,2,4‐triazolate‐1*N*‐oxide (DNTO^−^), and 3,4,5‐trinitropyrazolate‐1*N*‐oxide (TNPO^−^), affording their corresponding CMOFs in good yields. For the metals (Figure 3A,C), two primary differences (salt of the same metal and different metals) were considered. For the synthesis of CMOF(DNT), the metal source can be Cu(BF_4_)_2_, Cu(NO_3_)_2_, CuSO_4_, and other Cu^2+^ salts. In addition, not just Cu^2+^, but also Co^2+^, Zn^2+^, and Fe^2+^ were used to generate the corresponding CMOFs: CMOF(CuDNT), CMOF(CoDNT), CMOF(ZnDNT), and CMOF(FeDNT), respectively. All the CMOF structures were characterized using elemental analysis, IR spectroscopy, and PXRD. Single‐crystal X‐ray diffraction results (Figure 3B,C) indicated that all the azolate anions were encapsulated within the cationic frameworks and were not coordinated with the metals.

Owing to the large amounts of single‐crystal X‐ray data obtained, we performed a comprehensive comparative study of the coordination bond lengths and void sizes of the CMOFs. As shown in Figure 3D, the average values of the coordination bond lengths of the different metals were similar; however, the magnitude of the difference between the maximum and minimum values significantly differed. Unlike Co^2+^, Fe^2+^, and Zn^2+^, which have similar bond lengths, the difference in bond lengths for the six coordination bonds of the Cu^2+^ was large (> 0.35 Å). Conversely, Zn^2+^ had the largest difference (0.119 Å). The longest coordination bonds of Cu^2+^ were all Cu─O bonded with water; however, for the other metals, the difference between Cu─O and Cu─N bonds was smaller, with the N bonds less distinct. As shown in Figure 3E, the pore sizes of individual CMOFs were also investigated. The results revealed that the change in anion type could still regulate the pore size of the CMOFs despite the same ligand, and the pore sizes varied from 8.4 to 10.4 Å. The difference in the pore sizes of the same ions for different metals was small (0.3 Å). These results demonstrate that azolate and metal exchange can effectively regulate the structure of CMOFs, which may result in different physical and chemical properties.

### 2.3 Physical and Energetic Performances

The encapsulation of a series of polynitroazolate anions indicated that the resulting CMOFs could be applied as high‐energy‐density materials. These materials play indispensable roles in solid propellants, space exploration, and defense technologies. Thus, their physicochemical properties, particularly energy and safety, were investigated. The results are summarized in **Table** **1**.

**Table 1:** Energetic properties of different EMOFs.

Compd.	T_d_ ^a)^	ρ^b)^	N%^c)^	O+N%^d)^	IS^e)^	FS^f)^	−∆_c_U^g)^	∆_f_H_o_ ^h)^	P^i)^	VOD^j)^	Vo^k)^	‐∆_ex_U_o_ ^l)^
CMOF(CuNTT)	221	1.762	52.60	68.21	8	54	7616	1622	24.74	7816	747	4976
CMOF(CuDNT)	257	1.840	48.92	70.43	21	144	7662	1873	27.08	7966	709	5193
CMOF(CuTNP)	250	1.839	43.75	70.67	23	108	9302	2227	30.68	8282	702	5835
CMOF(CuNTTO)	242	1.751	48.47	72.97	15	72	9075	2720	31.01	8455	776	6816
CMOF(CuDNTO)	212	1.792	46.39	71.45	14	84	8122	1310	26.17	7888	734	4923
CMOF(CuTNPO)	212	1.827	41.70	71.97	13	72	10 699	3500	35.72	8735	714	7375
CMOF(CoDNT)	232	1.816	49.23	70.87	14	84	7933	1558	26.41	7716	621	5546
CMOF(ZnDNT)	228	1.822	48.80	70.26	12	192	8349	1861	27.58	7877	616	6056
CMOF(FeDNT)	234	1.792	47.69	71.16	14	96	8739	2189	27.59	8035	701	5789
NMOF(CuDNT)	282	2.155	41.51	62.58	3	12	4744	1169	23.78	7463	572	3513
CMOF(CuNO_3_)^m)^	223	1.64	53.56	67.70	16	112	8275	1651	17.4	6810	626	4562
TNT^n)^	290	1.65	18.50	60.79	15	353	–	−55.5	20.5	7176	620	5099
RDX^o)^	210	1.81	37.84	81.06	7.5	120	–	70.3	34.5	8861	785	5845
CL‐20^p)^	221	2.04	38.36	82.18	4	48		365		9700	715	6534

^a)^ The onset decomposition temperature (DSC, °C);

^b)^ Density measured from gas pycnometer(g cm^−3^);

^c)^ Oxygen content;

^d)^ Combined oxygen and nitrogen content;

^e)^ Impact sensitivity (J);

^f)^ Friction sensitivity (N);

^g)^ Experimental determined (oxygen bomb calorimetry) contant volume energy of combustion (kJ mol^−1^);

^h)^ Experiment determined (back‐calculated from ‐∆_c_ *U*) enthalpy of formation (kJ mol^−^);

^i)^ Detonation pressure (GPa);

^j)^ Detonation velocity (m s^−1^);

^k)^ Volume of gases after detonation (L kg^−1^);

^l)^ Heat of detonation (kJ kg^−1^);

^m)^ Properties of CMOF(CuNO_3_) are taken from ref. [5a]

^n)^ Properties of TNT are taken from ref. [17]

^o)^ Properties of RDX are taken from ref. [5a]

^p)^ Properties of CL‐20 are taken from ref. [17] All detonation parameters including detonation pressure, detonation velocity, explosion temperature, volume of gases after detonation and heat of detonation, were calculated by EXPLO5 v6.01.

Density is one of the most important properties of high‐energy‐density materials; therefore, the densities of all the CMOFs were measured using a gas pycnometer. All the values were within the range of 1.751–1.840 g cm^−3^. Their densities were higher than that of CMOF(CuNO_3_) (1.64 g cm^−3^), which is the most typical energetic CMOF with inorganic nitrate as the counter anion. CMOF(CuTNP) exhibited the highest density of 1.840 g cm^−3^, which was higher than that of CMOF(CuTNPO) (1.827 g cm^−3^), primarily due to its smaller voids. In addition, these CMOFs possessed high *N+O* contents of 70.26–72.97%, which were high values among all metal‐containing compounds and higher than that of CMOF(CuNO_3_) (67.7%). As a critical performance metric, the heat of detonation (*Q*) was calculated with EXPLO5 v6.01 using the experimentally determined (back‐calculated from −Δ_c_ *U*) enthalpy of formation and densities. The calculated heats of detonation were within the range of 4923–7375 kJ kg^−1^ and were superior to that of CMOF(CuNO_3_) (4562 kJ kg^−1^).^[^ ^16^ ^]^ In particular, the heats of detonation of CMOF(CuNTTO) and CMOF(CuTNPO) were as high as 6816 and 7375 kJ kg^−1^, respectively, which were even higher than that of the most powerful explosive, CL‐20 (6534 kJ kg^−1^).

In addition to energy, safety is an important indicator of their potential application. To evaluate the thermal stabilities of the energetic CMOFs, TGA/DSC measurements were performed. TGA revealed a gradual weight loss between 50 and 150 °C, which was attributable to the release of both the solvated and coordinated water molecules (see supporting information). Subsequently, the major weight loss in the temperature range of 180–280 °C indicated the decomposition of nitroazolate anions, atrz ligands, and the collapse of the frameworks. The onset decomposition temperatures of the CMOFs were from 212 to 257 °C. In addition, the mechanical sensitivity of these energetic CMOFs toward impact and friction was measured using standard BAM methods, and the results indicated that all the CMOFs (IS: 8–23 J; FS: 54–192 N) were more mechanically insensitive than RDX (IS: 7.5 J; FS: 120 N) and CL‐20 (IS: 4 J; FS: 48 N). These above results indicate that encapsulating polynitroazolate anions can provide high energy and safety, demonstrating that the resulting CMOFs can be used as high‐energy‐density materials.

### 2.4 Comparison to Analogs with Azolate Serving as the Ligand

**Figure 4:**
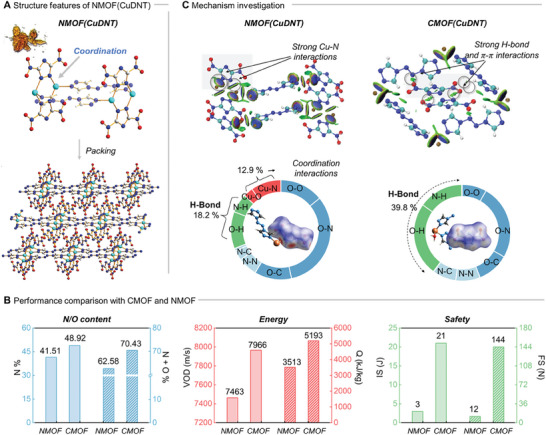
Structural and performance comparison between NMOF(CuDNT) and CMOF(CuDNT). A) Coordination structures of NMOF(CuDNT); B) N/O content, energy, and safety of NMOF(CuDNT) and CMOF(CuDNT). C) Non‐covalent interactions, Hirshfeld surface, and the percent contribution of the individual atomic contacts to the Hirshfeld surfaces of NMOF(CuDNT) and CMOF(CuDNT).

To confirm the superiority of azolates as counter anions, analogs with azolates as ligands were investigated. Still using CMOF(CuDNT) as the model, its analog NMOF(CuDNT) with a formula of [Cu_2_(atrz)_2_(DNT)_2_]_n_ was obtained as orange plate crystals after changing the reaction temperature to 80 °C (**Figure** **4**A). Single‐crystal X‐ray diffraction results indicated that DNT^−^ was coordinated with Cu^2+^, and each Cu^2+^ adopted a four‐coordinate octahedral geometry, coordinating with both atrz and DNT^−^. DNT^−^ formed four coordination bonds with Cu^2+^, spanning from 1.93 to 2.80 Å. Owing to the coordination of DNT^−^ with Cu^2+^, NMOF(CuDNT) did not exhibit a cationic framework structure.

The physical and energetic performances of CMOF(CuDNT) and NMOF(CuDNT) were compared (Figure 4B). Although NMOF(CuDNT) had a higher density (2.155 vs 1.840 g cm^−3^), the effective energetic components were fewer because of the lower coordination number of Cu^2+^. This result was supported by the higher *N* and *N+O* contents in CMOF(CuDNT). Furthermore, the detonation velocity and heat of detonation values of CMOF(CuDNT) were higher than those of NMOF(CuDNT) (*VOD*: 7966 and 7463 m s^−1^, *Q*: 5193 and 3513 kJ kg^−1^, respectively). Thus, CMOF(CuDNT) exhibited higher energy than NMOF(CuDNT). In addition, CMOF(CuDNT) exhibited better safety, with impact and friction sensitivities of 21 J and 144 N, respectively, while NMOF(CuDNT) was more sensitive to external stimuli (*IS*: 3 J, *FS*:12 N). Thus, CMOFs, with azolate anions encapsulated within the framework, exhibit higher energy and safety than those with azolate anions serving as ligands.

The intermolecular noncovalent interactions between CMOF(CuDNT) and NMOF(CuDNT) were investigated to explain the higher safety of CMOF(CuDNT). As shown in Figure 4C, DNT^−^ in NMOF(CuDNT) was primarily surrounded by strong coordination bonds. Conversely, DNT^−^ in CMOF(CuDNT) was surrounded by hydrogen bonds and π–π interactions, which are weaker than coordination bonds, explaining its better safety against impact and friction.^[^ ^18^ ^]^ To further quantify the differences in the intermolecular interactions of the DNT anions, Hirshfeld surfaces were calculated using the CrystalExplorer program.^[^ ^19^ ^]^ The interaction between DNT^−^ and Cu^2+^ in CMOF(CuDNT) was 0%, whereas that in NMOF(CuDNT) was 12.9%. Additionally, hydrogen bonding in CMOF(CuDNT) was more present than in NMOF(CuDNT) (39.8% > 18.2%), further demonstrating the effectiveness of our strategy in encapsulating azolate anions in high‐energy‐density materials with good energy and safety.

## 3 Conclusion

This study successfully achieved the encapsulation of azolates, which existed as counter anions rather than ligands, within CMOFs for the first time. These CMOFs exhibited good generalizability and included six types of azolate anions (3,4,5‐trinitropyrazolate, 3,5‐dinitrotriazolate, 5‐nitrotetrazolate, and their N‐oxides) and four types of metals (Fe, Co, Cu, and Zn). Even with the same metal and organic ligands, the framework configurations and pore environments varied with changes in the encapsulated anions. Moreover, the CMOFs exhibited good energetic performance. In particular, CMOF(CuTNPO) possessed an extremely high heat of detonation (7375 kJ kg^−1^), which was higher than that of the most powerful explosive, CL‐20, demonstrating its potential as a high‐energy‐density material. Compared to NMOF(CuDNT) with azolate as a ligand, CMOF(CuDNT) exhibited higher *N+O* content, heat of detonation, and mechanical insensitivity values. This study enriches the structural diversity of CMOFs and promotes the development of framework materials for more potential applications, such as high‐energy‐density materials.

[CCDC 1431829 1431830 1431832 1431833 1477835 1477836 1477888 1480889 1480890 2374747 contains the supplementary crystallographic data for this paper. These data can be obtained free of charge from The Cambridge Crystallographic Data Centre via www.ccdc.cam.ac.uk/data_request/cif.

## Conflict of Interest

The authors declare no conflict of interest.

## Supporting information



Supporting Information

## Data Availability

The data that support the findings of this study are available in the supplementary material of this article.
