# Supplementary material for: Encapsulating Azolates Within Cationic Metal–Organic Frameworks for High‐Energy‐Density Materials
Source: Adv Sci (Weinh). 2024 Sep 27;12(1):2409093. doi: 10.1002/advs.202409093 (PMC11714169; doi:10.1002/advs.202409093)
Supplement: Supplementary file 1 — Supporting Information [file ADVS-12-2409093-s001.pdf]

ADVANCED SCIENCE
Open Access

Supporting Information

for *Adv. Sci.*, DOI 10.1002/advs.202409093

Encapsulating Azolates Within Cationic Metal–Organic Frameworks for High-Energy-Density Materials

Ning Ding, Chaofeng Zhao, Jichuan Zhang, Yao Du, Qi Sun, Shenghua Li* and Siping Pang**

Supporting Information

Encapsulating Azolates within Cationic Metal–Organic Frameworks for High-Energy-Density Materials

Ning Ding, Chaofeng Zhao, Jichuan Zhang, Yao Du, Qi Sun, Shenghua Li* and Siping Pang**

School of Materials Science & Engineering, Beijing Institute of Technology, Beijing 100081, China.

E-mail: sunqi@bit.edu.cn *(Qi Sun)*; lishenghua@bit.edu.cn (*Shenghua Li*); pangsp@bit.edu.cn (*Siping Pang*).

Table of Contents

1. General methods
2. Safety precautions
3. Experimental procedures
4. Crystal structures
5. Measurement of friction and impact sensitivities
6. Energetic performance calculation
7. Photos of CMOF(CuDNT) prepared with different metal salts
8. Thermal stability
9. Schematic representation of pore sizes

1. General Methods

All reagents were purchased from Energy Chemical of analytical grade and were used as supplied, if not stated otherwise. The decomposition temperatures were obtained on a thermogravimetric analysis and differential scanning calorimeter (Mettler Toledo -TGA/DSC) at a scan rate of 10 °C min^{-1} in closed Al containers with a nitrogen flow of 50 mL min^{-1}. IR spectra were recorded using KBr pellets for solids on a Thermo Nicolet iS10 spectrometer. Elemental analyses were carried out on a vario EL III CHNOS elemental analyzer.

2. Safety Precautions

All the new EMOFs in this manuscript are powerful explosives, and should be handled with extreme care using the best safety practices.

3. Experimental procedures

3.1 Preparation of various nitrogen-rich azoles based ammounium salts.

Ammonium 3,4,5-trinitropyrazolate (TNPA)

According to the previous synthetic procedure,[1] 3,4,5-Trinitropyrazole (203 mg,1 mmol) and aqueous ammonia (61 mg, 1 mmol) were placed in water(5 mL). The mixture was stirred at room temperature for 0.5 h, water was removed under reduced pressure, the product was isolated as a light yellow solid. Elemental analysis calcd (%) for $C_3H_4N_6O_6$ (220.10): C 16.37, H 1.83, N 38.18; found: C 16.39, H 1.67, N 37.90; IR (KBr pellet, λ, cm^{-1}): 3161, 1763, 1664, 1519, 1397, 1129, 850, 825.

Ammonium 3,4,5-trinitropyrazole-1-olate (TNPOA)

According to the previous synthetic procedure,[2] Ammonium 3,4,5-trinitropyrazolate (3.302 g, 15 mmol) was dissolved in H_2O (45 mL). The solution was heated to 55 °C, and Oxone (27.675 g, 45 mmol) was added in small portions over 45 min. After the solution was stirred at 55 °C for three days, the reaction mixture was cooled to room temperature followed by the addition of sufficient water to dissolve all solids. Concentrated sulfuric acid (12 mL) was added dropwise to the solution with stirring at 0 °C. The yellow solution which was obtained was extracted with portions of ethyl ether until the aqueous phase was colorless. The ethyl ether extracts were combined and dried over anhydrous sodium sulfate followed by neutralization with an excess of aqueous ammonia solution at 0 °C. The reaction mixture was evaporated by blowing air, and then dried under vacuum getting the resulting yellow solid. Elemental analysis (%) calcd for $C_3H_4N_6O_7$ (236.10): C, 15.26; H, 1.71; N, 35.60; found: C, 15.39; H, 1.46; N, 35.25; IR (KBr pellet, λ, cm^{-1}): 3138, 1629, 1547, 1512, 1420, 1399, 1334, 1226, 1089, 937, 845, 796, 762.

Ammonium 3,5-dinitro-1,2,4-triazolate (DNTA)

According to the previous synthetic procedure,[3] 3,5-dinitro-1,2,4-triazole was synthesized, employing the same method like getting Ammonium 3,4,5-trinitropyrazolate, Ammonium 3,5-

dinitro-1,2,4-triazolates (DNTA) was gained as brownish red solid. Elemental analysis (%) calcd for $C_2H_4N_6O_4$ (176.03): C, 13.64; H, 2.29; N, 47.73; found: C, 13.39; H, 2.46; N, 47.55; IR (KBr pellet, λ, cm^{-1}):3312, 3140, 3021, 2833, 1556, 1498, 1392, 1357, 1303, 1102, 1055, 849, 651.

Ammonium 3,5-dinitro-1,2,4-triazole -1-olate (DNTOA)

According to the previous synthetic procedure,[2] Ammonium 3,5-dinitro-1,2,4-triazolate -1-olate was synthesized as faint yellow solid. Elemental analysis (%) calcd for $C_2H_4N_6O_5$ (192.09): C, 12.51; H, 2.10; N, 43.75; found: C, 12.33; H, 2.24; N, 43.49; IR (KBr pellet, λ, cm^{-1}): 3151, 1546, 1382, 1351, 1233, 1110, 1045, 840, 697, 552.

Ammonium 5-Nitrotetrazolate (NTTA)

According to the previous synthetic procedure,[4] Ammonium 5-Nitrotetrazolate was synthesized as white solid. Elemental analysis (%) calcd for $C_2H_4N_6O_2$ (132.08): C 9.09, H 3.05, N 63.63; found: C 8.63, H 3.22, N 63.38; IR (KBr, pellet, λ, cm^{-1}): 3267, 3162, 3006, 2847, 1704, 1554, 1448, 1417, 1318, 1182, 1167, 1063, 1038, 835, 670.

Ammonium 5-Nitrotetrazole -1-olate (NTTOA)

According to the previous synthetic procedure,[5] Ammonium 5-Nitrotetrazole -1-olate was synthesized as faint yellow solid. Elemental analysis (%) calcd for $C_2H_4N_6O_3$: C, 8.11; H, 2.72; N, 56.75. Found: C, 8.09; H, 2.68; N, 56.30; IR (KBr, pellet, λ, cm^{-1}): 3162, 1664, 1546, 1472, 1428, 1318, 1235, 1089, 1061, 997, 846, 783, 663.

3.2 Synthesis of various Nitrogen-rich heterocyclic CMOFs

Atrz (0.050 g, 0.3 mmol), $Cu(NO_3)_2$ (0.17 g, 0.9 mmol), and Ammonium fully C-nitrated azolate salts (0.6 mmol) were added into 20 ml water. The mixed solution was stirred for 1 h, and then the resulting solution was filtered. 2-5days later. The single crystals suitable for X-ray diffraction were obtained by slow evaporation in a glass vial within several days.

CMOF(TNP): Yield: 75% based on the atrz. Elemental analysis (%) calculated for $CuC_{14}H_{12}N_{26}O_{14}$ (M=832): C, 20.19; H, 1.44; N, 43.75. Found: C, 20.01; H, 1.34; N, 44.06; IR

(KBr pellets, λ, cm^{-1}): 3161, 3132, 3114, 1548, 1493, 1397, 1370, 1335, 1181, 1039, 887, 621, 552.

CMOF(TNPO): Yield: 63% based on the atrz. Elemental analysis (%) calculated for $CuC_{14}H_{13.04}N_{26}O_{16.52}$ (M=873): C, 19.24; H, 1.49; N, 41.69. Found: C, 20.03; H, 1.37; N, 42.08; IR (KBr pellets, λ, cm^{-1}): 3138, 1639, 1547, 1512, 1399, 1334, 1226, 1186, 1044, 876, 842, 764, 622, 552.

CMOF(DNT): Yield: 75% based on the atrz. Elemental analysis (%) calculated for $CuC_{12}H_{12}N_{26}O_{10}$ (M=744): C, 19.35; H, 1.62; N, 48.93. Found: C, 19.89; H, 1.41; N, 50.23. IR (KBr pellets, λ, cm^{-1}): 3382, 3159, 3114, 1667, 1546, 1495, 1384, 1351, 1320, 1297, 1216, 1182, 1050, 885, 839, 698, 649, 622, 555.

CMOF(DNTO): Yield: 61% based on the atrz. Elemental analysis (%) calculated for $CuC_{12}H_{16}N_{26}O_{12.29}$ (M=785): C, 18.34; H, 2.04; N, 46.37. Found: C, 18.89; H, 1.98; N, 47.09. IR (KBr pellets, λ, cm^{-1}): 3158, 3114, 1662, 1547, 1495, 1383, 1351, 1233, 1182, 1048, 841, 699, 623, 555.

CMOF(NTT): Yield: 82% based on the atrz. Elemental analysis (%) calculated for $CuC_{10}H_{16}N_{26}O_{8}$ (M=687): C, 17.34; H, 2.31; N, 52.61. Found: C, 17.85; H, 2.17; N, 53.43. IR (KBr pellets, λ, cm^{-1}): 3538, 3359, 3138, 3122, 3063, 2965, 1666, 1630, 1541, 1503, 1442, 1416, 1342, 1317, 1189, 1064, 1050, 884, 836, 703, 671, 625, 556.

CMOF(NTTO): Yield: 74% based on the atrz. Elemental analysis (%) calculated for $CuC_{10}H_{8}N_{26}O_{10.5}$ (M=740): C, 16.22; H, 1.08; N, 49.19. Found: C, 16.76; H, 1.17; N, 49.88. IR (KBr pellets, λ, cm^{-1}): 3172, 3108, 1664, 1548, 1516, 1501, 1470, 1459, 1422, 1384, 1316, 1224, 1188, 1047, 1038, 1007, 842, 782, 705, 622, 551.

CMOF(CoDNT): Yield: 77% based on the atrz. Elemental analysis (%) calculated for $CoC_{12}H_{12}N_{26}O_{10}$ (M=739.4): C, 19.35; H, 1.62; N, 48.93. Found: C, 19.89; H, 1.41; N, 50.23. IR (KBr pellets, λ, cm^{-1}): 3114, 3119, 1690, 1665, 1548, 1495, 1436, 1413, 1385, 1353, 1299, 1216, 1185, 1116, 1044, 876, 843, 696, 649, 620, 552.

MOF(ZnDNT): Yield: 75% based on the atrz. Elemental analysis (%) calculated for $CuC_{12}H_{12}N_{26}O_{10}$ (M=746): C, 19.35; H, 1.62; N, 48.93. Found: C, 19.89; H, 1.41; N, 50.23. IR (KBr pellets, λ, cm^{-1}): 3147,3119, 1691, 1547, 1494, 1385, 1352, 1298, 1217, 1186, 1115, 1043, 697, 649, 620, 552.

MOF(FeDNT): Yield: 74% based on the atrz. Elemental analysis (%) calculated for $CuC_{12}H_{12}N_{26}O_{10}$ (M=736): C, 19.35; H, 1.62; N, 48.93. Found: C, 19.89; H, 1.41; N, 50.23. IR (KBr pellets, λ, cm^{-1}): 3139, 3119, 1548, 1495, 1385, 1353, 1299, 1215, 1186, 1117, 1042, 875, 843, 696, 649, 620, 551.

NMOF(DNT): The same feed as CMOF (CuDNT) was placed at 80°C for growth. Yield: 56% based on the atrz. Elemental analysis (%) calculated for $C_8H_4Cu_2N_{18}O_8$ (M=607): C, 11.86; H, 0.65; N, 41.51. Found: C, 12.03; H, 1.01; N, 41.29. IR (KBr pellets, λ, cm^{-1}): 3417, 3139, 1516, 1380, 1344, 1287, 1193, 1101, 1043, 1001, 886, 814, 678, 669, 612.

4. Crystal Structures

X-ray crystallography details: Crystals were mounted on an Enraf-Nonius CAD4 four-circle diffractometer using graphite-monochromated Mo Ka radiation (λ = 0.71073 Å) at 103-273 K. Corrections for Lorentz and polarization effects and for absorption (ψ scan) were applied. The structure was solved by direct methods using SHELXS-97 and refined by full-matrix least-squares calculation on F2 with SHELXL-97. All non-hydrogen atoms were refined anisotropically. All hydrogen atoms were placed in calculated positions and were assigned fixed isotropic thermal parameters at 1.2 times the equivalent isotropic U of the atoms to which they were attached and allowed to ride on their respective parent atoms. The contributions of these hydrogen atoms were included in the structure-factor calculations.

Table S1. Crystallographic data and strucutre refinement parameters of different MOFs

Crystal	MOF(DNT)	MOF(CoDNT)	MOF(ZnDNT))	MOF(FeDNT)	MOF(DNTO)
Formula	$C_{12}H_{12}Cu$ $N_{26}O_{10}$	$C_{12}H_{12}Co$ $N_{26}O_{10}$	$C_{12}H_{12}Zn$ $N_{26}O_{10}$	$C_{12}H_{12}Fe$ $N_{26}O_{10}$	$C_{12}H_{16}Cu$ $N_{26}O_{10}$
Formula mass	744.03	739.41	745.9	736.3	784.33
Crystal system	Triclinic	Triclinic	Triclinic	Triclinic	Triclinic
Space group	P-1(2)	P-1(2)	P-1(2)	P-1(2)	P-1(2)
Z	1	1	1	1	1
a (Å)	6.1505	5.9427	5.9479	5.9098	7.9470
b (Å)	9.8159	10.171	10.156	10.2744	8.7744
c (Å)	11.1766	11.343	11.423	11.4694	10.8289
α (°)	100.315	103.093	103.237	104.112	100.554
β (°)	94.103	96.081	96.323	97.237	105.051
γ (°)	90.043	90.482	90.472	90.602	92.454
Volume ($Å^3$)	662.09	663.7	667	669	713.61
D_{calc}(gcm^{-3})	1.866	1.850	1.856	1.827	1.826
Temperature (K)	103	173	173	173	173
F(000)	375.0	373.0	376	372	397.3
h, k, l	7, 12, 13	7,12,13	7,12,13	7,12,13	9, 10, 13
μ (cm-1)	0.930	0.752	1.025	0.668	0.874
$R_1[I > 2\sigma(I)]$	0.0340	0.0376	0.0399	0.0373	0.0320
Reflections collected	0.0340(2318)	0.0376(1877)	0.0399(1897)	0.0373(1878)	0.0320(2118)
Completeness to theta full	0.999	0.985	0.989	0.983	0.982
wR_2(all data)	0.0828(2606)	0.1232(2496)	0.0982(2435)	0.0776(2421)	0.0672(2560)
S on$F2$	1.097	1.031	1.050	1.031	1.022

Table S2. Crystallographic data and strucutre refinement parameters of different MOFs

Crystal	MOF(NTT)	MOF(NTTO)	MOF(TNP)	MOF(TNPO)	NMOF(DNT)
Formula	$C_{12}H_{16}Cu$ $N_{26}O_8$	$C_{20}H_{38}Cu_2$ $N_{52}O_{23}$	$C_{14}H_{12}Cu$ $N_{26}O_{14}$	$C_{14}H_{13.03}Cu$ $N_{26}O_{16.52}$	$C_8H_4Cu_2$ $N_{18}O_8$
Formula mass	692.04	1502.12	832.04	873.34	607.37
Crystal system	Triclinic	Monoclinic	Triclinic	Triclinic	Monoclinic
Space group	P-1(2)	P 21/C	P-1(2)	P-1(2)	P 21/n
Z	1	4	1	1	4
a (Å)	6.4975	6.5567	6.6828	15.6192	10.6862
b (Å)	8.9481	30.772	10.2011	15.8132	11.2531
c (Å)	11.0062	14.8654	11.1333	14.8195	16.0686
α (°)	90.278	90	79.339	90	90
β (°)	94.806	111.173	82.794	121.669	105.155
γ (°)	96.506	90	84.146	90	90
Volume (Å^3)	633.48	2796.8	737.57	3115.2	3115.2
D_{calc}(gcm^{-3})	1.814	1.784	1.873	1.862	2.163
Temperature (K)	103	173	173	171	273
F(000)	351.0	1528.0	419.0	1760.6	1865.1
h, k, l	8, 11, 13	8, 40, 19	8, 12, 13	18,19,17	13,14,20
μ (mm^{-1})	0.959	0.885	0.856	0.821	2.374
R_1[I > 2σ(I)]	0.0327	0.0499	0.0469	0.0372	0.0408
Reflections collected	0.0327(2266)	0.0499(4029)	0.0469(1841)	0.0372(2067)	0.0408(2909)
Completeness to theta full	0.998	0.982	0.981	0.985	0.996
wR_2(all data)	0.0821(2478)	0.1162(6394)	0.0799(2628)	0.1000(2811)	0.1275(3812)
S on*F2*	1.132	1.013	1.010	1.038	1.029

5. Measurement of friction and impact sensitivities

Impact sensitivity: the impact sensitivity was tested on a type 12 tooling according to "up and down" method (Bruceton method). A 2.5 kg weight was dropped from a set height onto a 20 mg sample placed on 150 grit garnet sandpaper. Each subsequent test was made at the next lower height if explosion occurred and at the next higher height if no explosion happened. 50 drops were made from different heights, and an explosion or non-explosion was recorded to determine the results. RDX was considered as a reference compound, the impact sensitivity of RDX is 7.4 J.

Friction sensitivity: the friction sensitivity was tested on a FSKM-10 BAM friction apparatus. RDX was also used as a reference compound, and its friction sensitivity is 110 N.

6. Energetic performance calculation

6.1 Measurement of contant volume energy of combustion of energetic MOFs

For the calorimetric measurements, a Parr 6200 bomb calorimeter (static jacket) with a 6510 water handling system for the combustion of energetic MOFs was used. The calorimeter was calibrated by the combustion of certified benzoic acid (about 1.0 g, pellet) in an oxygen atmosphere at a pressure of 3.05 MPa. The samples (~0.3 g each) were prepared and placed in combustion pots, which were subsequently burned in a 3.05 MPa atmosphere of pure oxygen. The experimentally determined energy of combustion was obtained as the averages of three single measurements. The experimental results for the constant volume combustion energy ($\Delta_c U$) of energetic MOFs are listed in **Table 1**.

6.2 Calculation of enthalpies of formation of various energetic MOFs

The constant-volume combustion energies ($\Delta_c U$) for the desolvated MOFs were measured by an oxygen bomb calorimeter, along with MOF(Cu) and RDX as reference materials. The enthalpy of formation ($\Delta_c H^o$) was calculated from Δ_cU and a correction for change in gas volume during combustion was included (**eq 1**). Hess's Law as applied in thermochemical eqs **2**, the standard enthalpies of formation ($\triangle_f H°$) of these energetic MOFs, MOF(Cu) and RDX were back calculated from the heats of combustion on the basis of combustion eqs **3-14**, and known standard heats of formation for metallic oxide, water and carbon dioxide [$\triangle_f H°$(CuO, s) = -156.06 kJ mol^{-1};$\triangle_f H°$(CoO, s) = -237.74 kJ mol^{-1}; $\triangle_f H°$(Fe_2O_3, s) = -825.50 kJ mol^{-1}; $\triangle_f H°$(ZnO, s) = -350.46 kJ mol^{-1}; $\triangle_f H°$(CO_2, g) = -393.51 kJ mol^{-1}; $\triangle_f H°$(H_2O, l) = -285.83 kJ mol^{-1}].[6]

1) $\Delta_c H^0 = \Delta_c U + \Delta nRT$
2) $\Delta_f H^0$ (reactant, s)= $\sum \Delta_f H^0$ (product, s) + $\sum \Delta_f H^0$ (product, l) + $\sum \Delta_f H^0$ (product, g) -$\Delta_c H^0$ (reactant, s)
[Δn=Δni(products,g)-Δnj(reactants,g),Δni orΔnj is the total amounts of gases in the products or reactants]

3) MOF (Cu): $C_{12}H_{12}CuN_{26}O_6$ (s) + 12.5O_2 (g) → 12CO_2 (g) + 6H_2O (l)+ CuO (s)+13N_2 (g)

4) RDX: $C_3H_6N_6O_6$ (s) + 1.5O_2 (g) → 3CO_2 (g) + 3H_2O (l)+ 3N_2 (g)

5) CMOF (CuDNT): $C_{12}H_{12}CuN_{26}O_{10}$ (s) + 10.5O_2 (g) → 12CO_2 (g) + 6H_2O (l)+ CuO (s)+13N_2 (g)

6) CMOF (CoDNT): $C_{12}H_{12}CoN_{26}O_{10}$ (s) + 10.5O_2 (g) → 12CO_2 (g) + 6H_2O (l)+ CoO (s)+13N_2 (g)

7) CMOF (ZnDNT): $C_{12}H_{12}ZnN_{26}O_{10}$ (s) + 10.5O_2 (g) → 12CO_2 (g) + 6H_2O (l)+ ZnO (s)+13N_2 (g)

8) CMOF (FeDNT): $C_{12}H_{12}FeN_{26}O_{10}$ (s) + 10.5O_2 (g) → 12CO_2 (g) + 6H_2O (l)+ Fe_2O_3 (s)+13N_2 (g)

9) CMOF (DNTO): $C_{12}H_{16}CuN_{26}O_{12.29}$ (s) + 10.355O_2 (g) → 12CO_2 (g) + 8H_2O (l)+ CuO (s)+13N_2 (g)

10) CMOF (TNP): $C_{14}H_{12}CuN_{26}O_{14}$ (s) + 10.5O_2 (g) → 14CO_2 (g) + 6H_2O (l)+ CuO (s)+13N_2 (g)

11) CMOF (TNPO): $C_{14}H_{12}CuN_{26}O_{16}$ (s) + 9.5O_2 (g) → 14CO_2 (g) + 6H_2O (l)+ CuO (s)+13N_2 (g)

12) CMOF (NTT): $C_{10}H_{12}CuN_{26}O_6$ (s) + 10.5O_2 (g) → 10CO_2 (g) + 8H_2O (l)+ CuO (s)+13N_2 (g)

13) CMOF (NTTO): $C_{10}H_{19}CuN_{26}O_{11.5}$ (s) + 9.5O_2 (g) → 10CO_2 (g) + 9.5H_2O (l)+ CuO (s)+13N_2 (g)

14) NMOF (CuDNT): $C_8H_3CuN_{18}O_8$ (s) + 6O_2 (g) → 8CO_2 (g) + 2H_2O (l)+ 2CuO (s)+ 9N_2 (g)

Scheme S1. Combustion reaction and Hess's Law of various energetic MOFs and RDX

6.3 Calculation of detonation properties of various energetic MOFs

We adopted two different methods for the calculation of detonation properties of various MOFs: (i) our developed method on the basis of the empirical Kamlet formula[7,8]; ii) the commercial program EXPLO5 v6.01. Beacuse the deviation of detonation properities coming from these two methods are smaller,[9] we employed our method to calculate the detonation properties of MOFs containing Zn and Co(**Scheme S2**), and employed the commercial program EXPLO5 v6.01 to calculate the detonation properties of MOFs containing Fe and Cu.

Based on the detonation reactions of energetic MOFs, their detonation properties were evaluated by the empirical Kamlet formula,[3] as follows:

$V_D = 1.01(NM^{1/2}Q^{1/2})^{1/2}\ (1+1.30\rho)$

$P = 1.55\ NM^{1/2}Q^{1/2}\rho^2$

Q= -[$\Delta_f H$(detonation products)- $\Delta_f H$(explosive)]/formula weight of explosive

In the Kamlet equations, V_D represents detonation velocity (km s^{-1}) and P is detonation pressure (GPa), ρ is the density of explosive (from gas pycnometer, g cm^{-3}). Φ, N, M and Q are characteristic parameters of an explosive. N is the moles of detonation gases per gram of explosive, M is the average molecular weight of these gases and Q is the heat of detonation (kcal kg^{-1}). $\Delta_f H$(explosive) is the experiment determined (back-calculated from $-\Delta_c U$) enthalpy of formation of energetic MOF.

15) CMOF (CoDNT): $C_{12}H_{12}CoN_{26}O_{10}$ (s) $\longrightarrow$ $2CO_2$ (g) + $6H_2O$ (l)+ Co (s)+$13N_2$ (g) + 10C (s)

16) CMOF (ZnDNT): $C_{12}H_{12}ZnN_{26}O_{10}$ (s) $\longrightarrow$ 10C (s) + $6H_2O$ (l)+ Co (s)+$40/3N_2$ (g) + $4/3NH_3$(g)

Scheme S2. Detonation reactions of energetic MOFs containing Zn and Co.

7. Photos of CMOF(CuDNT) prepared with different metal salts

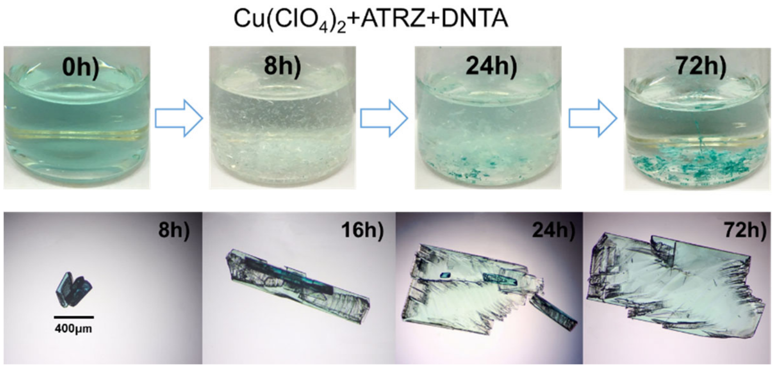

Figure S1. Preparation of CMOF(CuDNT) with $Cu(ClO_4)_2$.

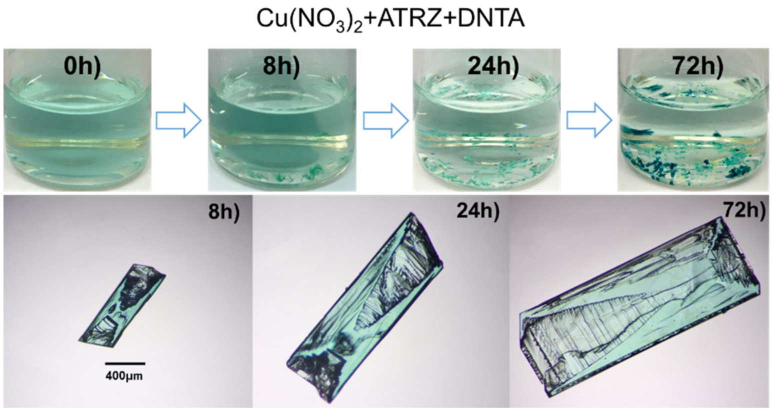

Figure S2. Preparation of CMOF(CuDNT) with $Cu(NO_3)_2$.

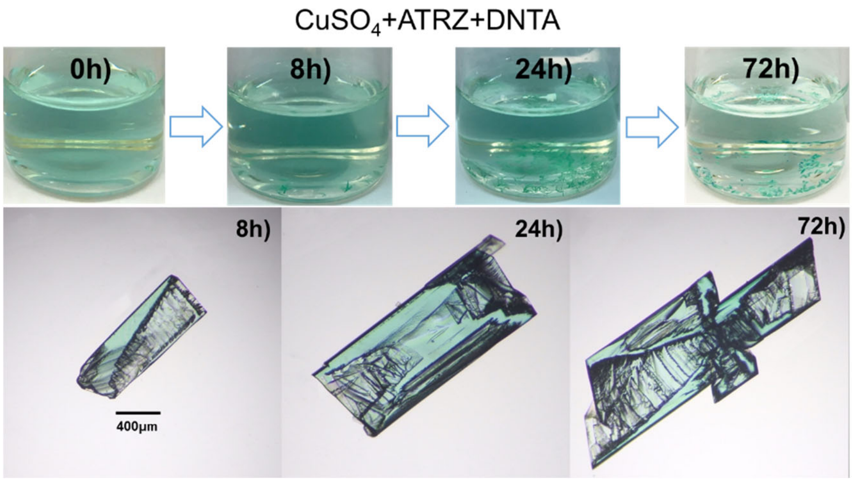

Figure S3. Preparation of CMOF (CuDNT) with $CuSO_4$.

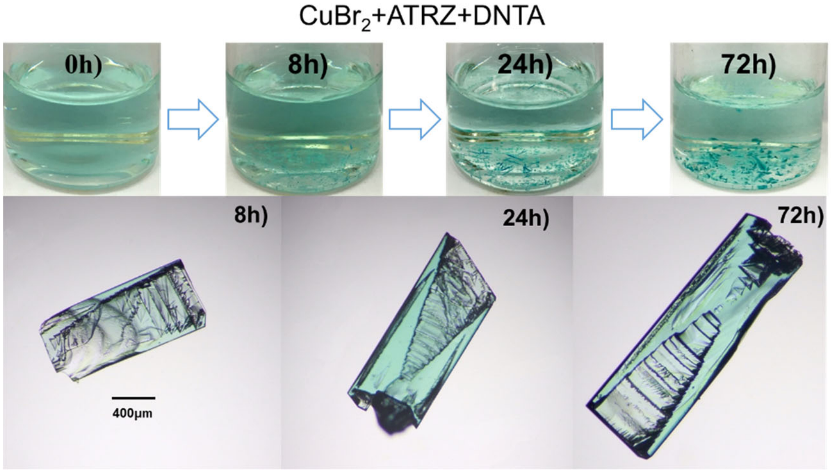

Figure S4. Preparation of CMOF (CuDNT) with $CuBr_2$.

8. Thermal stability

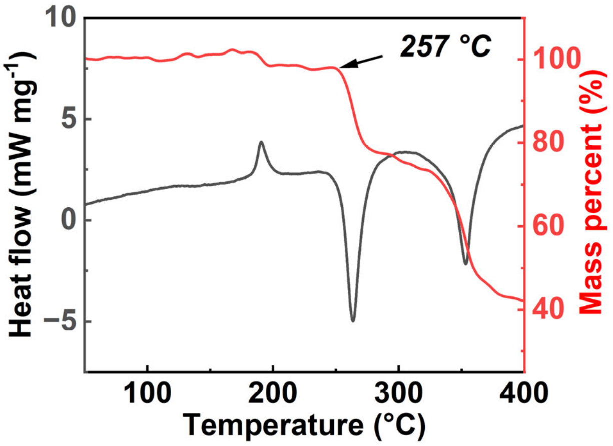

Figure S5. TG-DSC plot for CMOF(CuDNT).

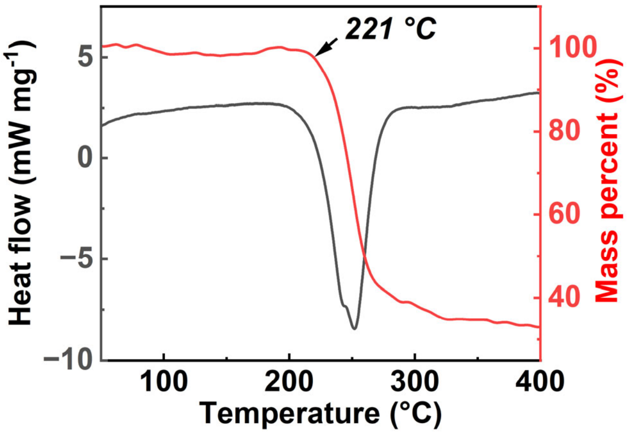

Figure S6. TG-DSC plot for CMOF(CuNTT).

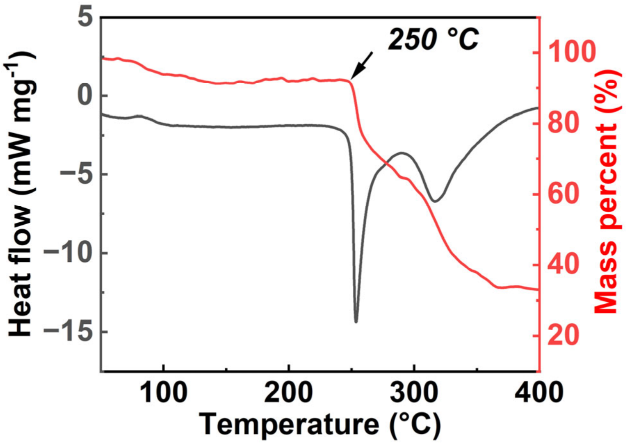

Figure S7. TG-DSC plot for CMOF(CuTNP).

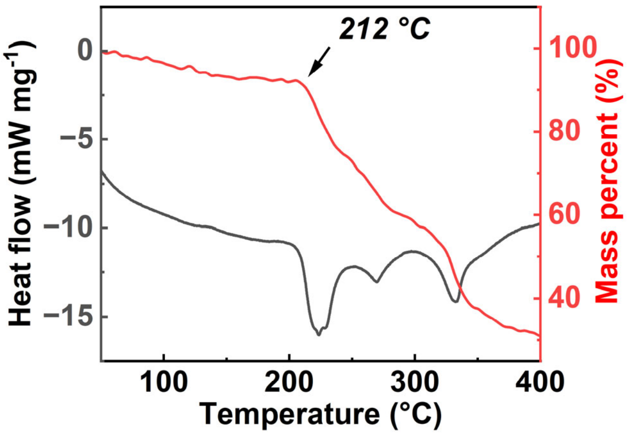

Figure S8. TG-DSC plot for CMOF(CuDNTO).

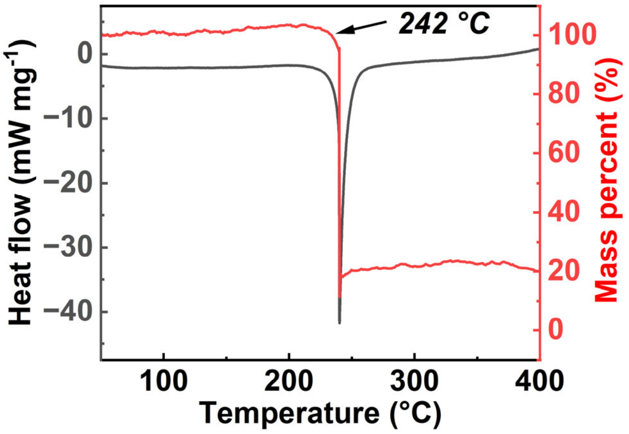

Figure S9. TG-DSC plot for CMOF(NTTO)

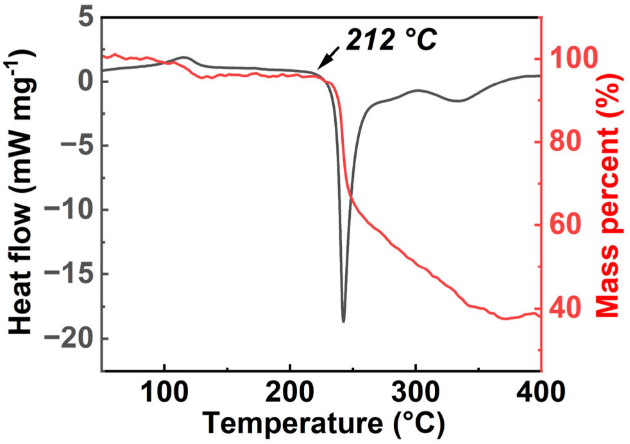

Figure S10. TG-DSC plot for CMOF(CuTNPO).

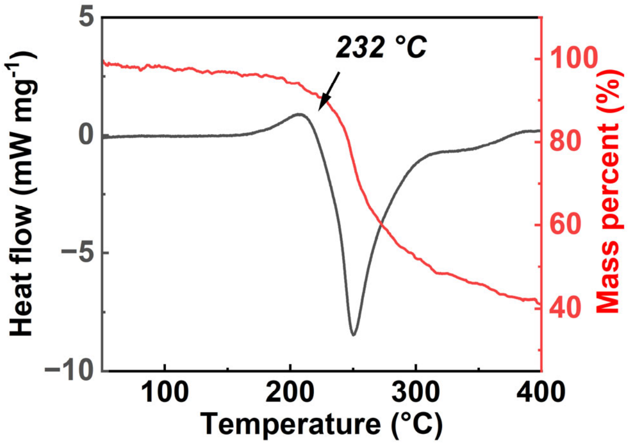

Figure S11. TG-DSC plot for CMOF(CoDNT).

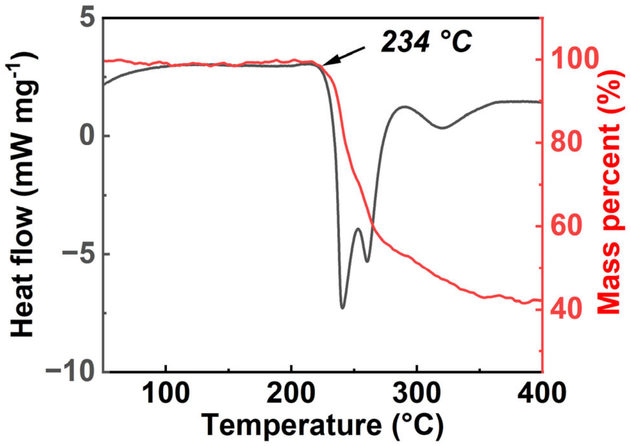

Figure S12. TG-DSC plot for CMOF(FeDNT).

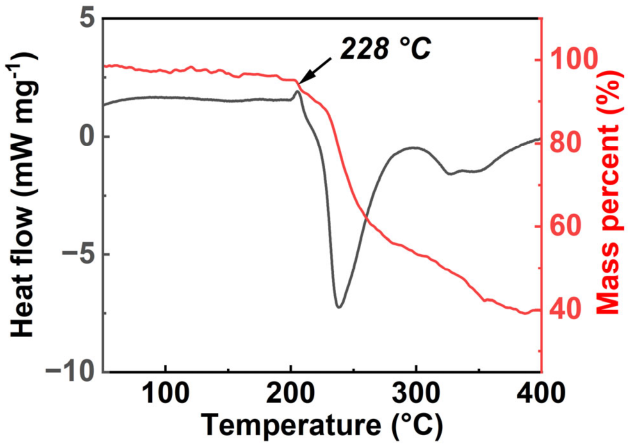

Figure S13. TG-DSC plot for CMOF(ZnDNT).

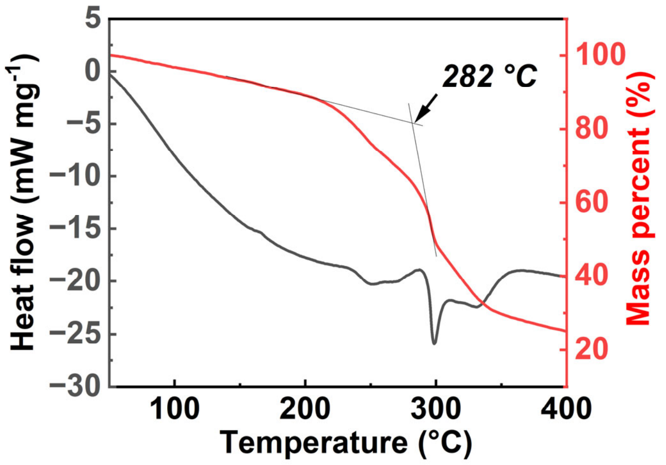

Figure S14. TG-DSC plot for NMOF(CuDNT).

9. Schematic representation of pore sizes

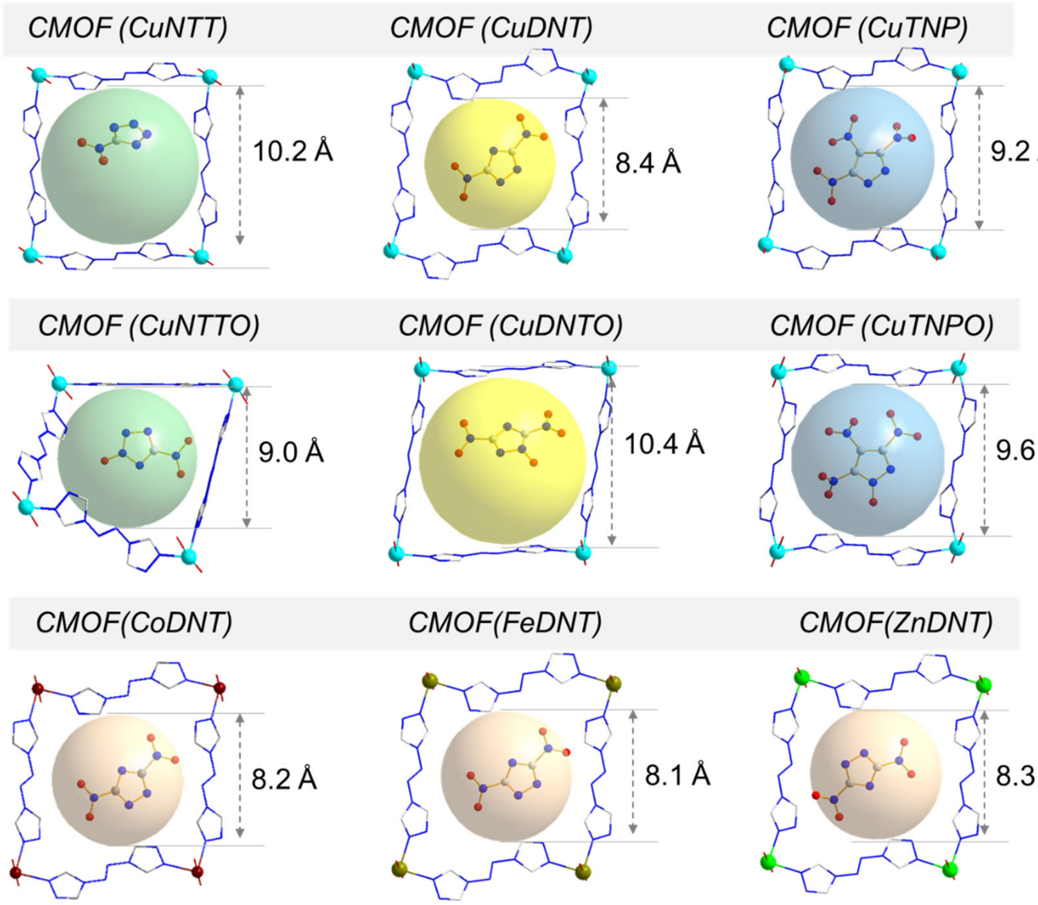

Figure S15. Structures of different CMOFs. The large spheres indicate the size of the voids.

REFERENCE

(1) Hervé, G; Roussel, C; Graindorge, H. Selective Preparation of 3,4,5-Trinitro-1H-Pyrazole:A Stable All-Carbon-Nitrated Arene. *Angew. Chem. Int. Ed.* **2010**, 49, 3177 –3181.

(2) Zhang, Y.; Parrish, D. A; Shreeve, J. M. Synthesis and properties of 3,4,5-trinitropyrazole-1-ol and its energetic salts. *J. Mater. Chem.* **2012**, 22, 12659–12665.

(3) Haiges, R.; Bélanger-Chabot, G.; Kaplan, S. M.; Christe, K. O. Synthesis and structural characterization of 3,5-dinitro-1,2,4-triazolates. *Dalton Trans*. **2015**, 44, 2978–2988.

(4) Klapötke, T. M.; Mayer, P.; Sabaté, C. M.; Welch, J. M.; Wiegand, N.; Simple, Nitrogen-Rich, Energetic Salts of 5-Nitrotetrazole. *Inorg. Chem.* **2008**, 47, 6014-6027.

(5) Göbel,M.; Karaghiosoff,K.; Klapötke, T. M.; Piercey, D. P.; Stierstorfer, J. Nitrotetrazolate-2N-oxides and the Strategy of N-Oxide Introduction. *J. Am. Chem. Soc.* **2010**, 132, 17216–17226.

(6) Lide, D. R. Standard Thermodynamic Properties of Chemical Substances. *CRC Handbook of Chemistry and Physics, Internet Version* **2007**, 87th ed.; Taylor and Francis: Boca Raton, FL, **2007**.

(7) Kamlet, M. J.; Jacobs, S. J. The Chemistry of Detonations. 1. A Simple method for Calculation Detonation properities of CHNO Explosives. *J. Chem. Phys*. **1968**, 48, 23-35.

(8) Wang, Y.; Zhang, J.; Su, H.; Li, S.; Zhang, S.; Pang, S. A Simple Method for the Prediction of the Detonation Performances of Metal-Containing Explosives. *J. Phys. Chem. A.* **2014**, 118, 4575-4581.

(9) Klapötke, T. M.; Martin, F. A.; Mayr, N. T. Stierstorfer J. Synthesis and Characterization of 3, 5 - Diamino - 1, 2, 4 - triazolium Dinitramide. *Z. Anorg. Allg. Chem.* **2010**, 636, 2555-2564.
